# Recent trends in the chromatographic analysis of volatile flavor and fragrance compounds: Annual review 2020

**DOI:** 10.1002/ansa.202000158

**Published:** 2021-02-04

**Authors:** Stefan Louw

**Affiliations:** ^1^ Department of Chemistry and Biochemistry University of Namibia Windhoek Namibia

**Keywords:** Electronic nose, electronic tongue, gas chromatography, headspace analysis, ion mobility spectrometry, solvent assisted flavor evaporation

## Abstract

The chromatographic analysis of volatile flavor and fragrance compounds is performed routinely in several industries and in many fields of scientific research. Typical applications include food‐, environmental‐, essential oil‐ and cosmetics analysis. Even though the analysis of flavors and fragrances have become increasingly standardized during the past decade, there are still a large variety of techniques that can be used for their extraction, chemical analysis, and sensory analysis. Moreover, there are certain less commonly used techniques that are now being used with increased frequency and that are showing the potential of being used as alternatives to the existing standard techniques. In this annual review, the techniques that were most commonly used in 2020 for the investigation of these volatile compounds are discussed. In addition, a number of emerging trends are discussed, notably the use of solvent assisted flavor evaporation (SAFE) for extraction, GC ion mobility spectrometry (IMS) for volatile compound analysis and electronic senses, that is, E‐noses and E‐tongues, for sensory analysis. Miscellaneous hyphenated techniques, advances in stationary phase chemistry and a number of interesting applications are also highlighted.

## 1 INTRODUCTION

During the past decade, the analysis of flavor and fragrances have become increasingly standardized. Particularly, a number of guidelines for the analysis of these compounds were published by working groups of the International Organisation of the Flavor Industry (IOFI).^1^, ^2^, ^3^, ^4^, ^5^, ^6^ The most recent guidelines are for the gas chromatography‐mass spectrometry (GC‐MS) and liquid chromatography‐mass spectrometry (LC‐MS) identification of flavor and fragrance compounds^6^ and for the quantification of the constituents of flavors, fragrances, and essential oils.^5^ The other guidelines include the quantitative GC analysis of volatile flavoring substances,^1^ as well as specific guidelines for the quantitative analysis of these substances using either GC‐MS in single ion monitoring (SIM) mode,^4^ or GC coupled to a flame ionization detector (FID) and employing predicted relative response factors.^2^ In addition, guidelines were published specifically for the solid‐phase micro‐extraction (SPME) GC analysis of volatile flavor compounds.^3^ Notably, all these guidelines are exclusively for chromatographic methods of analysis.

Typically, volatile organic compounds (VOCs) are either extracted directly from a sample or from the sample's headspace, prior to analysis. Alternatively, the headspace gas of a sample can be analyzed directly, by injecting it into an instrument's inlet using, for example, a gas‐tight headspace syringe. VOCs can be extracted directly from a sample using a number of different techniques, including steam distillation (SD),^7^ simultaneous distillation extraction (SDE),^8^ solvent‐assisted flavor extraction (SAFE),^9^ stir‐bar sorptive extraction (SBSE),^10^ and direct‐immersion solid‐phase microextraction (DI‐SPME).^11^ Headspace volatiles, on the other hand, are extracted using solventless sorptive extraction techniques, either by static headspace sampling, for example, using headspace (HS) SPME or headspace sorptive extraction (HSSE) (using a coated stir‐bar), or by dynamic headspace sampling, for example, dynamic headspace (DHS) extraction or purge‐and‐trap headspace (P&T‐HS) extraction.^12^ Characterization of the volatiles is subsequently performed by, for example, GC‐MS, GC‐FID, GC ion‐mobility spectrometry (IMS), and so on, while sensory analysis can be performed using, for example, GC olfactometry (GC‐O) and/or electronic senses (electronic nose (E‐nose) and/or electronic tongue (E‐tongue)). In many instances, the resulting data is interpreted with the help of a number of different chemometric techniques. Quite often, a collaborative approach is adopted, where a series of different techniques are used to generate complementary data, to provide a more comprehensive understanding of, for example, perceived flavor differences of different food samples.^13^

In this annual review, recent trends in the chromatographic analysis of flavor and fragrance compounds, published in 2020, are discussed, highlighting the techniques most commonly used, as well as emerging techniques. In addition, a number of interesting applications are discussed.

## 2 TRENDS IN THE CHROMATOGRAPHIC ANALYSIS OF VOLATILE FLAVOR AND FRAGRANCE COMPOUNDS

### 2.1 Gas chromatography‐mass spectrometry

When searching on SCOPUS for, for example, volatile flavor chromatography within the fields TITLE‐ABSTRACT‐KEYWORDS and limiting the search to 2020/2021, 424 documents are found (search performed on January 1, 2021). When repeating the search, but also adding the search terms *gas chromatography mass spectrometry* the number of documents reduced to 343. Hence, 81% of publications on the chromatographic analysis of volatile flavor compounds published in 2020 reports the use of GC‐MS. Performing the same search sequence, but replacing the search term *flavor* with *fragrance*, 84% of the publications involves the use of GC‐MS.^1^ Of course it is not surprising, since GC‐MS is still regarded to be the “gold standard” for the analysis of volatile flavor and fragrance compounds and it is indeed routinely used for this purpose.^14^ A number of different headspace sampling techniques, one direct extraction technique, sensory analysis techniques, and comprehensive two‐dimensional gas chromatography (GC×GC) techniques used in combination with MS were reported in 2020 and are discussed in this section, highlighting emerging trends and interesting applications.

#### 2.1.1 Headspace sampling techniques

**FIGURE 1:**
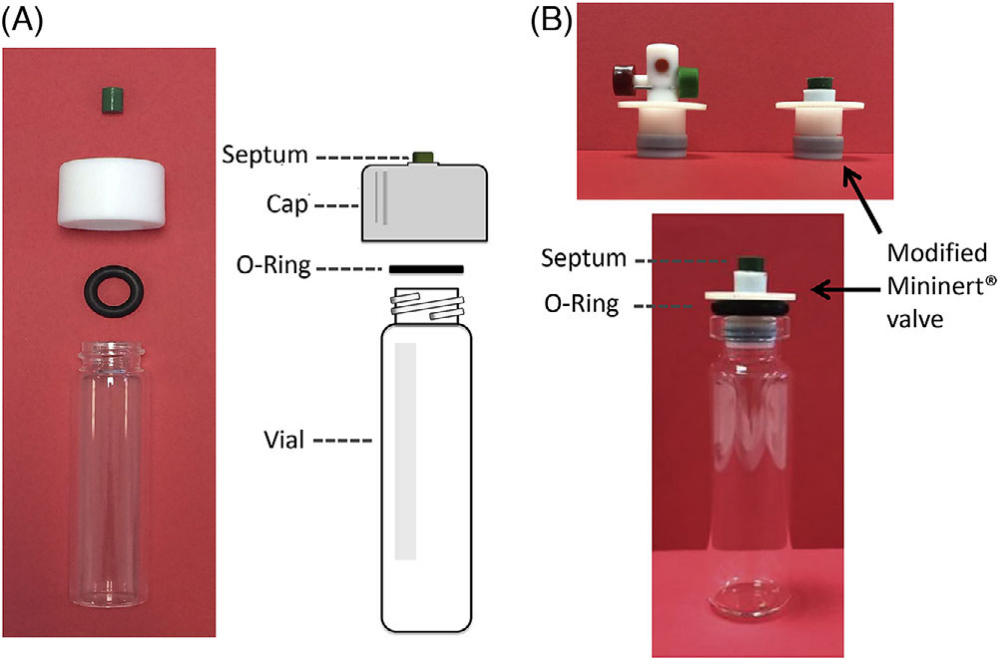
Examples of vials and caps used for Vac‐HS‐SPME: (A) O‐ring seal with molded screw cap design and (B) modified Mininert® valve design (Image reprinted from ref. 31, Copyright (2017), with permission from Elsevier)^31^

Liberto et al.^12^ recently published a book chapter that covers all of the different headspace sampling techniques comprehensively. In 2020, the analysis of headspace volatile flavor and fragrance compounds using GC‐MS was dominated by the use of HS‐SPME, particularly in food analysis^15^, ^16^, ^17^, ^18^, ^19^, ^20^, ^21^, ^22^, ^23^, ^24^, ^25^, ^26^, ^27^ and environmental analysis applications.^28^, ^29^, ^30^ In addition, a number of variations on how HS‐SPME sampling is performed were reported. A noteworthy emerging technique is vacuum‐assisted (Vac) HS‐SPME, where the extraction is performed under vacuum in order to improve the extraction of semi‐volatile compounds that typically have a low affinity for the headspace, without the need to use high extraction temperatures. Other advantages of the technique include shorter extraction times and high extraction efficiencies. Headspace sampling under vacuum is facilitated by the use of a variety of suitable sample containers, notably regular headspace vials equipped with O‐ring sealed caps with septa (Figure 1), capable of maintaining low pressures for at least 24 h and that opens up the possibilities for automation of the technique. In the sampling procedure, the vial is first evacuated using a vacuum pump connected to a needle that is inserted into the septum. Once the desired pressure level has been reached, a liquid sample can be introduced into the vial through the septum using a syringe, while a solid sample would already be present prior to evacuating the vial. Subsequently, the HS‐SPME extraction is performed in the usual way while the vial is still under vacuum.^31^ Recent food analysis applications of Vac‐HS‐SPME‐GC‐MS include the analysis of extra‐virgin olive oil^17^ and soybean oil,^18^ while in one fragrance analysis application it was used to discriminate the frankincense resins of different *Boswellia* spp. by comparing their volatile and semi‐volatile fractions.^32^ In the latter study, ground resin samples were weighed into headspace vials prior to connecting the vacuum system. To minimize the loss of the volatile compounds, the vials were cooled to below 0°C during the evacuation stage. The vials were then incubated at the relevant sampling temperature which was followed by HS‐SPME sampling of the compounds. The use of different sample amounts (5, 40, and 100 mg), sampling temperatures (50 and 80°C), and sampling durations (5, 15, 30, and 60 min) were evaluated. The optimal Vac‐HS‐SPME conditions were found to be the sampling of 100 mg of resin at 80°C for 15 min. This method was directly compared to a conventional HS‐SPME method for the analysis of the different resins. The use of the Vac‐HS‐SPME resulted in a pronounced improvement of the recovery of the groups of semi‐volatile compounds, i.e. the diterpene hydrocarbons, the oxygenated diterpenes, and most of the oxygenated sesquiterpenes. A number of these compounds serve as markers for the discrimination of the different frankincense resins. The optimized Vac‐HS‐SPME was combined with fast GC using ionic liquid‐based GC stationary phases for improved selectivity, which could successfully be applied to discriminate the different resins that were investigated. This combination resulted in a fast, simple, automated method suitable for routine analysis.^32^

Another variation of how SPME is used is the dual‐fiber SPME technique (also called two‐fiber SPME), which was used for the analysis of volatile compounds in traditional Chinese dry‐cured ham.^33^ In this approach, two fibers are simply exposed to the same sample simultaneously and subsequently, the volatiles are desorbed one by one from each of the fibers and trapped using a cooled injection system (CIS), prior to GC‐MS analysis. The dual‐fiber approach resulted in a greater coverage and higher extraction efficiency of the headspace volatiles and could be used to distinguish between three different grades of Chinese dry‐cured hams.^33^

An adapted SPME device worth highlighting is the SPME Arrow. The insufficient extraction phase volume employed in regular SPME is one of the major disadvantages of this technique when compared to, for example, SBSE.^34^ The SPME Arrow, introduced in 2015,^35^ has a thicker extraction phase, with a phase volume that is 24 times more than in conventional SPME.^36^ It has for instance been shown that the former technique provides improved detection of a wider range of compounds than the latter.^37^ A more detailed account of the SPME Arrow technique can be found in a recent review.^34^ In one of the most recent reports of the use of this technique, a method was developed that facilitated the quantitative GC‐MS analysis of 82 aroma compounds, namely esters, alcohols, fatty acids, aldehydes, ketones, furans, pyrazines, sulfur compounds, phenols, terpenes, and lactones that were present in the headspace of a Chinese liquor (Baijiu). The use of a new type of SPME Arrow fiber (divinylbenzene (DVB)/carboxen (CAR), wide range (WR)/ polydimethylsiloxane (PDMS) 120 μm fiber) was directly compared to regular SPME with a DVB/CAR/PDMS 50/30 μm fibre. The number of volatile compounds detected using the two techniques did not differ significantly, but the SPME Arrow yielded higher sensitivity than SPME. Naturally this is expected, since the SPME Arrow has a higher volume of extraction phase.^36^

**FIGURE 2:**
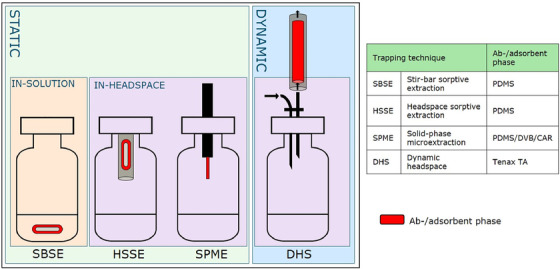
Comparison of HSSE, SBSE, SPME, and DHS for the analysis of flavor compounds (Image reprinted from ref. 38, an open‐access article under a Creative Commons license)^38^

Diez‐Simon et al^38^ compared the headspace sampling of food flavorings using HSSE, SBSE, DHS, and SPME, followed by GC‐MS analysis (Figure 2). The techniques were evaluated for their comprehensiveness and repeatability. It was revealed that a significantly higher number of constituents could be detected when using SBSE. However, SPME and DHS proved to be the most suitable techniques for the extraction of sesquiterpene and monoterpene hydrocarbons, respectively. Therefore these two techniques will also be suitable if the targeted analysis of the respective compound types would be desired. It was also concluded that SBSE and SPME were the techniques that provided the best repeatability for the analysis of the water‐soluble flavor compounds analyzed in the study. It is worthwhile noting that these conclusions are based on the particular samples analyzed in the study, that is, Maxagusto process flavors. In addition, HSSE and SBSE were performed using PDMS coated stir bars, while the SPME fiber that was used was a PDMS/DVB/CAR fiber and in the DHS extractions a Tenax TA sorbent was used. Nonetheless, HSSE and DHS still extracted mixtures of volatiles with very similar compositions.^38^

An interesting application where HSSE was used in combination with GC×GC‐time‐of‐flight mass spectrometry (TOFMS), is where the chemical fingerprints of the “emotional body odor” of humans were determined. This was achieved by studying the variation in the chemicals present in human sweat during different emotional states, i.e. fearful, happy and neutral. The sweat was collected using absorbent pads attached to the inside of the participants’ t‐shirts, and a number of different experiments were conducted where the different emotional states were induced. The volatile compounds present in the sweat were subsequently extracted from the pads using HSSE. This was performed by placing a sweat pad and PDMS coated stir‐bar into a glass bottle, with the stir‐bar being suspended against the side of the bottle (not touching the pad), using a magnet attached to the outside of the bottle. The extraction was then performed at 60°C for 2 h. The volatiles were thermally desorbed into the GC inlet and analyzed by GC×GC‐TOFMS. Multivariate statistical analysis of the data was used to test three models, namely fearful versus neutral, happy versus neutral, and happy versus fearful emotional states. Using this approach, the fearful state could be clearly distinguished from the neutral state. Although the happy state was found to be chemically significantly different from the other states, there was some overlap with both the neutral and fearful state. Moreover, it could be determined that the classes of chemicals associated with these differences are linear aldehydes, ketones, and esters, as well as cyclic compounds containing five rings.^39^

A number of dynamic headspace sampling approaches have recently been used for different applications, including environmental analysis,^40^, ^41^, ^42^ food flavor and fragrance analysis,^43^ and studying animal behavior.^44^ Notably, in one study, a low‐cost air sampling pump named a “FloPump” was compared to the commercially available Supelco equivalent, used for dynamic headspace sampling. The headspace volatiles were trapped onto volatile collection traps containing 20 mg of Porapak Q (Volatile Collection Trap LLC) prior to GC‐MS analysis. The analyses were performed on the headspace volatiles of a ripened guava fruit and a commercial perfume sample. It was found that the sampling efficiency of the low‐cost FloPump (ca. 86% cheaper than the Supelco pump) was comparable to the commercial alternative.^43^

An interesting example of a variation of dynamic headspace sampling is where semi‐volatile compounds were trapped in condensed water samples in portable dehumidifiers. This was performed in order to screen the semi‐volatile compounds present in indoor air in order to assess their potential impact on human health. Although the authors did not report this approach as being a dynamic headspace sampling technique *per se*, technically the dehumidifiers are serving the same purpose as a dynamic headspace sampling system, using the water as the trapping medium. Once trapped, the compounds were extracted from the water using solid‐phase extraction prior to GC‐TOF‐MS analysis. Using this approach a total of 141 semi‐volatile compounds could be identified in a number of indoor air samples, including several fragrance related potentially allergenic compounds.^42^

The alternative to static and dynamic headspace extraction techniques are techniques where the headspace gasses of a sample are simply transferred directly into an instrument for analysis. In one study, the use of high‐temperature headspace sampling in combination with GC‐MS was explored for the analysis of terpenoids and nicotine in plant materials and vaping products (used e.g. in E‐cigarettes). The headspace gasses were transferred directly into the GC injector using a 1 mL heated sample loop. The samples were dissolved in glycerol (headspace carrier liquid) and their headspace volatiles were sampled at 180°C in order to have conditions that correspond to what is encountered during the vaping process. The method could be used effectively to determine the terpenoid profiles and nicotine content of the samples under these conditions.^45^

Headspace hot injection and trapping (HS‐HIT) is a multiple headspace sample enrichment technique where the headspace gasses are also directly injected into the instrument. Specifically, multiple portions of the headspace gasses of a sample are injected into a thermal desorption unit (TDU) using a headspace syringe. The TDU is connected to a CIS where the volatiles from the repeated injections are trapped on Tenax TA sorbent prior to being introduced into a GC column for analysis. In a recent study, HS‐HIT was compared to static headspace sampling (SHS), HS‐SPME, and HS‐HIT‐SPME for the analysis of volatile compounds in kimchi, traditional Korean fermented vegetables. In the optimized HS‐HIT method, the TDU and CIS temperatures were maintained at 250°C and ‐40°C, respectively, and nine 1 mL aliquots of the sample's headspace were injected into the TDU. In the SHS approach, a single 1 mL aliquot of headspace gasses was analyzed by injecting it into a regular split/splitless injector operated in splitless mode at 250°C. The split/splitless injection system was also used for the HS‐SPME analyses, while the HS‐HIT‐SPME analyses were performed using the same TDU and CIS system that was used for the HS‐HIT analyses, however, only a single injection was performed. It was found that the highest number of compounds could be detected using the HS‐HIT technique and it also provided the highest recovery, based on the total peak area of all the detected constituents. However, although a higher number of the early eluting compounds could be detected when using HS‐HIT, a higher number of later eluting compounds could be detected using HS‐SPME. Since some of these compounds were not detected when using HS‐HIT, it was recommended that HS‐HIT and HS‐SPME should be used together as complementary techniques. On the other hand, since the sulfur compounds of interest in the analysis of kimchi are amongst the early eluting compounds, it could be concluded that HS‐HIT on its own should be a suitable technique for this application.^46^

#### 2.1.2 Analysis of volatiles extracted using solvent‐assisted flavor evaporation

**FIGURE 3:**
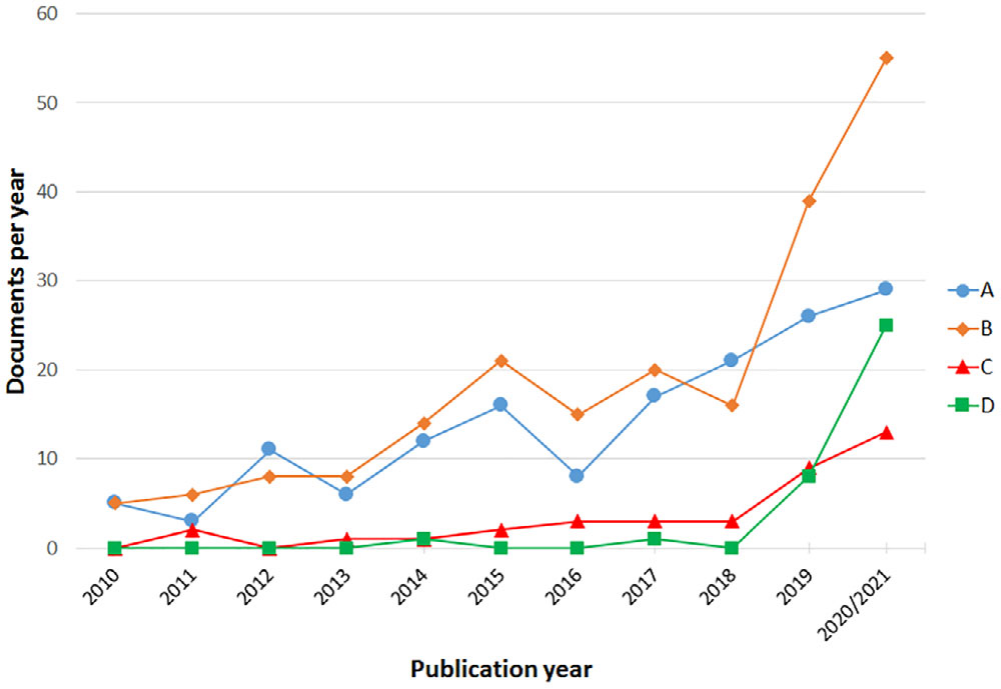
Search of literature in the SCOPUS database using the search terms *volatile* AND *flavor* AND *chromatography* within the fields TITLE‐ABSTRACT‐KEYWORDS and limiting the search to 2020/2021. The search terms (A) *solvent* AND *assisted* AND *evaporation*, (B), *electronic* AND *nose* (C), *electronic* AND *tongue* and (D) *ion* AND *mobility* was also added, respectively (Searches performed on 01/01/2021)

A technique that has become increasingly popular in recent years is the extraction of volatile flavor compounds using SAFE (Figure 3). The use of this technique was first reported in 1999 and was described as a “careful and direct isolation of aroma compounds from complex food matrices”.^9^ During the SAFE process volatile compounds are distilled from samples at low temperatures (40‐50°C) and under high vacuum conditions, thereby preventing the degradation of labile compounds during extraction.^9^ In 2020 it has been used for the extraction of volatile flavor and aroma compounds from different foods and beverages, including coffee,^47^ tea,^48^ beer,^7^, ^49^ wine,^50^ fruit and vegatables,^19^, ^26^, ^27^, ^51^, ^52^, ^53^, ^54^ fried food,^55^ butter,^25^ honey,^56^ liquor,^57^ vinegar,^58^ yeast extracts,^59^, ^60^ frying oil,^61^ millet,^62^ pericarp oil,^63^ and honeysuckle^64^ as well as for the extraction of Cembran pinewood odor compounds,^65^ prior to GC‐MS analysis.

Wieczorek et al.^27^ compared SPME, SAFE, and SDE for the analysis of the volatile flavor compounds in broccoli. The odor‐active compounds extracted with each of the techniques were first determined using GC‐O and then they were identified using GC‐MS. Compounds not detected or resolved in the GC‐MS analysis were characterized using GC×GC‐TOFMS. The GC×GC‐TOFMS volatile profiles of the three extracts were subsequently compared. It was found that the SAFE method yielded the highest number of potent odourants, followed by SDE and then SPME. However, the highly volatile compounds that co‐elute with the solvent used for the analysis of the SAFE and SDE extracts could be detected using SPME. Although SDE could be used to isolate many of the relevant aroma‐active compounds, a number of artifacts were also present due the high temperature used during extraction and hence the use of this technique is not preferred. It was concluded that the full aroma profile could be evaluated using both the SAFE and SPME techniques, since they complement each other.^27^

For similar reasons, SPME and SAFE were utilized in parallel in many other studies in order to get the best coverage of a wide enough range of the relevant volatiles.^19^, ^25^, ^26^, ^47^, ^49^, ^51^, ^52^, ^63^, ^64^ For instance, the use of five different extraction techniques, namely SBSE, P&T‐HS, SAFE, SPME, and DHS were compared for the flavor of compounds from tomato fruit. Again, it was found that the combination of SPME and SAFE provided the best coverage of the volatiles. Using this combination together with aroma extract dilution analysis (AEDA), the authors were able to determine that hexanal, 3‐hexenal, (*E*)‐2‐hexenal, 1‐penten‐3‐one, and guaiacol are key aroma‐active compounds in tomatoes.^52^

On the other hand, the extraction techniques, HS‐SPME, SAFE, and SD, were evaluated for their potential to be used to study the flavor stability of lager beer. It was found that HS‐SPME in combination with SD gave the best coverage of the relevant aging‐related volatile compounds. The highest number of aroma compounds could be extracted with SD, while the least number of compounds were extracted using SAFE. In addition, a qualitative evaluation using GC‐O showed that the aroma intensities of most of the aging‐related compounds extracted with the SAFE method were also the lowest, while those of the SD extract were the highest. Hence, SPME and SD were selected for further quantitative investigations using GC‐MS. It was found that the results can be influenced to a greater or lesser degree, by the extraction technique that was used, depending on the aging‐indicator compound analyzed as well as the sample matrix. Ultimately, the authors concluded that the non‐invasive SPME method would be the preferred one to use, since the breakdown and formation of certain important compounds can occur during the SD process.^7^

#### 2.1.3 Gas chromatography – olfactometry and electronic senses

The “gold standard” in instrumental sensory analysis, but employing the human nose, is GC‐O. In 2020 the use of GC‐O was reported almost exclusively within the food and beverage field^19^, ^25^, ^26^, ^47^, ^49^, ^55^, ^66^, ^67^, ^68^ and was discussed in reviews in the context of environmental analysis^69^ and recycled plastics.^70^ In a recent review, the application of GC‐O‐MS in food flavor analysis was described in detail,^66^ while the principles and practices of GC‐O and its application in food analysis are covered in a book chapter that was published in 2020.^71^

In a recent study, GC‐O was used to identify the odor‐active compounds that cause alcohol‐free beer to have a worty (unfermented beer like) flavor. These beers are known to contain undesirable malty and worty flavors, which has so far been contributed to the presence of so‐called Strecker aldehydes, e.g. 2‐methylbutanal, 3‐methylbutanal, methional, and phenylacetaldehyde. However, Piornos et al^49^ hypothesized that compounds other than the Strecker aldehydes contribute to the aroma of wort. First, the volatile compounds were extracted from the beer samples using the SAFE method and analyzed using GC‐O to determine the presence of different aroma‐active compounds. When some of the common highly volatile compounds were not detected the GC‐O experiments were repeated using HS‐SPME. Using a series of experiments, including AEDA, 27 odor‐active compounds could be identified and quantified in the beer, five of which were identified as the key aroma compounds, namely 5‐ethyl‐3‐hydroxy‐4‐methyl‐2(*5H*)‐furanone, (*E*)‐*β*‐damascenone, methional, 3‐methylbutanal, and phenylacetaldehyde. Thus it was confirmed that not only the Strecker aldehydes contribute to the wort aroma.^49^

A duo of emerging instrumental sensory analysis techniques that are used in parallel with chromatographic techniques for the analysis and evaluation of volatile flavor and fragrance compounds are electronic senses, namely E‐noses and E‐tongues. When repeating the SCOPUS search described in Section 2.1. with the search terms *electronic nose* and *volatile flavor chromatography*, 55 hits are listed for 2020/2021, while only 39 and 16 are listed for 2019 and 2018, respectively (Figure 3) (searched on 01/01/2021). That means more than double the number of documents were published in 2019 than what was published in 2018 and subsequently an increase is still observed in 2020/2021. Replacing *electronic nose* with *electronic tongue* in the search revealed that 13, 9 and 3 contributions were published in 2020/2021, 2019, and 2018, respectively (Figure 3) (searched on 01/01/2021).

An E‐nose typically consists of an array of metal oxide semiconductor sensors (MOSs), each capable of detecting olfactory cross‐sensitive information, with each sensor being capable of detecting certain groups of compounds, for example, aromatic compounds, ammonia as well as aromatic compounds, methane, sulfides, etc.^72^, ^73^, ^74^, ^75^, ^76^ Typically a headspace sample is analyzed directly with the E‐nose and then the volatile compounds are characterized using a separate technique such as HS‐SPME‐GC‐MS,^72^, ^73^, ^74^, ^75^, ^76^ but an e‐nose can also be directly hyphenated to a GC.^74^ An E‐tongue consists of sensors that can detect certain flavor characteristics including umami, saltiness, sourness, and aftertastes (eg, bitterness and astringency). Sample solutions are usually analyzed separately by the E‐tongue.^73^, ^75^, ^76^

In one study, HS‐SPME‐GC‐MS was used in combination with an electronic nose and an electronic tongue to distinguish between sausages prepared using traditional and conventional processing methods. The E‐nose that was used was a PEN‐3 system (Airsense Analytics GmbH, Schwerin, Germany) equipped with 10 metal oxide gas sensors that can detect 1, aromatic compounds, 2, nitrogen oxide, 3, ammonia as well as aromatic compounds, 4, hydrogen, 5, aromatic as well as aliphatic compounds, 6, methane, 7, sulfides, 8, alcohols, 9, aromatic compounds as well as organic sulfides and 10, alkanes. Headspace gasses of the sausage samples were analyzed directly using the E‐nose by injecting the gasses into the instrument. A SA402B E‐tongue (Insent Company, Atsugi‐Shi, Japan), equipped with 5 taste sensors, was used to analyze the taste attributes, (1) umami as well as richness (umami‐aftertaste), (2) saltiness, (3) sourness, (4) bitterness‐aftertaste, and (5) astringency‐aftertaste. Aqueous extracts of the sausage samples were analyzed using the E‐tongue. The results of the HS‐SPME‐GC‐MS analysis alone already revealed that the volatile compound composition of sausages processed by traditional and conventional methods differed significantly, since the total concentrations of aldehydes, ketones, phenols, and furans were found to be significantly higher in the traditional sausages. Using principal component analysis (PCA), both the volatile compound profiles and E‐nose data could be used to clearly distinguish between the sausages processed by the traditional and conventional methods, while the E‐tongue was unable to do so.^73^

Another study employed HS‐SPME‐GC‐MS and electronic nose data as well as data from a flash‐GC‐E‐nose (directly hyphenated) instrument, to investigate how the volatiles of Chinese jujubes are affected by different drying methods. A PEN‐3.5 E‐nose (Airsense Analytics, GmbH, Schwerin, Germany), equipped with sensors with very similar sensing capabilities as those of the PEN‐3 described above, was used for the E‐nose analysis of the headspace gasses of jujube samples. A flash‐GC coupled to a Heracles II E‐nose (Alpha M.O.S., Toulouse, France) was also used for the analysis of the dried jujube aroma, by injecting jujube sample headspace gasses directly into the GC inlet. Two columns were installed in the flash GC, one with a non‐polar stationary phase (MXT‐5) and one with an intermediate polarity stationary phase (MXT‐1701), and each column was connected to its own FID as well as to the E‐nose. The HS‐SPME‐GC‐MS data showed that the vacuum freeze‐dried jujubes had the highest total concentration of aroma compounds, while the instant controlled pressure drop dried samples had the highest number of compounds. PCA of the HS‐SPME‐GC‐MS and flash‐GC‐E‐nose data revealed that the volatile compound profiles of the jujubes dried using the remaining drying techniques, i.e. heat‐pump‐, infrared radiation‐, hot‐air‐, and vacuum‐dried jujubes, were similar. Using PCA and linear discriminant analysis, the data of the standalone E‐nose analysis could also be used to discriminate between the samples, with the regions in the PCA and LDA plots either overlapping or appearing close to each other for similar samples.^74^

#### 2.1.4 Comprehensive two‐dimensional gas chromatography

**FIGURE 4:**
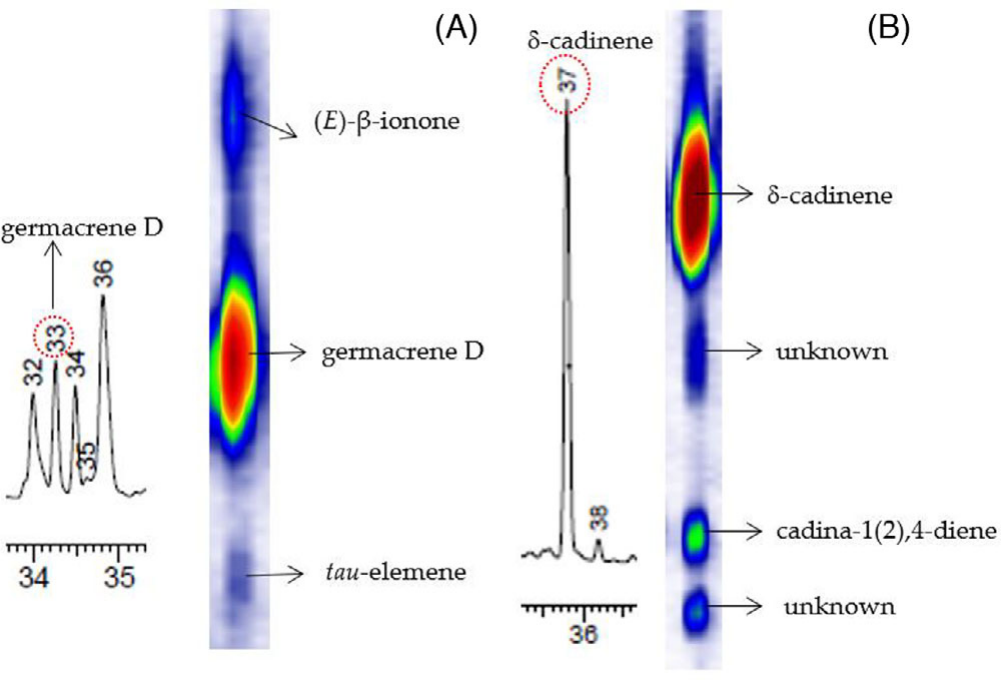
GC×GC separation of (A) germacrene D from *tau*‐elemene and of (B) δ‐cadinene from cadin‐1(2),4‐diene and two unknown constituents (only the relevant sections of the two‐dimensional contour plot is shown next to each section of the 1D chromatogram; the horizontal axis of the contour plot is the time axis of the first dimension, while the vertical axis represents the time axis of the second dimension). (Image reprinted from ref. 77, an open access article under a Creative Commons Attribution License)^77^

Since flavor and fragrance compounds are often present in highly complex samples, separations with sufficient resolution may frequently only be possible through the use of GC×GC analysis approaches, the most powerful techniques used for the separation of volatile and semi‐volatile compounds. A direct comparison of GC‐MS and GC×GC‐MS for the analysis of the essential oil produced from the leaves of *Varronia curassavica* Jacq. (syn. *Cordia verbenacea*), a medicinal plant found in Brazil, was performed. The main aim of the study was to compare the essential oil of three genotypes of *V. curassavica* as well as the seasonal variation of the oils that were produced from leaves harvested at different times of the year. Using a GC×GC‐MS equipped with a differential flow modulator, 135 compounds could be identified in the oils, while only 48 compounds could be identified using GC‐MS. This lead to the detection of many compounds that have not been identified in *V. curassavica* essential oil before. Examples of how GC×GC facilitated the separation of compounds co‐eluting when analyzed with one‐dimensional (1D) GC, was the separation of germacrene D from *tau*‐elemene and (E)‐β‐ionone as well as the separation of δ‐cadinene from cadin‐1(2),4‐diene and two unknown constituents (Figure 4). In addition to the detailed characterization of the *V. curassavica* essential oil, the active ingredient in the oil that is monitored by the pharmaceutical industry, α‐humulene, was quantified in the different oil samples. It was found that the concentration of this compound is influenced by both the genotype as well as the harvest season.^77^

In another study, a 1D‐GC‐MS method was developed for the detection of smoky off‐flavor compounds present in cacao, for the purpose of quality control. A top‐down approach was adopted where GC×GC‐TOFMS was first used to identify the compounds responsible for the off‐flavors and then a 1D‐GC‐MS method more suitable for routine analysis could be developed targeting the relevant compounds. In this approach, the detailed volatile profiles of a subset of smoky and non‐smoky cocao samples were determined using a GC×GC‐TOFMS equipped with a thermal modulator and a polar × semi‐polar column combination. The compounds responsible for the off‐flavors were then identified by using an advanced fingerprinting strategy to compare the 2D‐peak patterns. It was revealed that 56 compounds are more abundant in the smoky samples than in the non‐smoky sample, but only 10 of these compounds (naphthalene and a number of esters, alcohols, and phenols) could be expected to contribute to the smoky character of the cacao. Subsequently, the targeted 1D‐GC‐MS method could be developed, which involved further optimization of the HS‐SPME sample preparation step in order for the method to offer suitable sensitivity for the off‐flavor compounds of interest. Finally, the optimized HS‐SPME‐GC‐MS method was used to analyze all of the available smoky and non‐smoky samples. Using a number of chemometric techniques, the most relevant off‐flavor markers could be confirmed. These compounds were subsequently subjected to accurate quantitation in order to determine the limits at which they are allowed to be present, to define sample acceptability when performing these analyses for the purpose of quality control.^78^

The aroma profiles of fruit beers were compared using a combination of P&T‐HS and a low‐flow modulation GC×GC‐TOFMS approach. The P&T‐HS sampling was performed at 20°C, with a total purge volume of 750 mL, and the volatiles were trapped onto Tenax TA sorbent packed into thermal desorption tubes.^79^ The GC×GC was equipped with a differential flow modulator used in symmetrical configuration^80^ and the separations were performed using a non‐polar × mid‐polar column combination. In previous studies where differential flow modulators were used, the resulting flow (> 20 mL/min) was too high, which necessitated splitting the flow and hence only a fraction will enter the MS. In this study, an approach based on previous research^80^, ^81^ was used where the accumulation and injection phases of the modulation is optimized in such a way that the resulting overall flow entering the second dimension column and the MS is low enough (< 7 mL/min) so that no splitting of the flow is necessary, hence increasing sensitivity. The GC×GC‐TOFMS modulation conditions were optimised using a mixture of alkanes and working under isothermal conditions. In the optimised method, flowrates of 0.4 mL/min and 7 mL/min were used for the first and second dimensions, respectively. This method could subsequently be used successfully to discriminate between the different fruit beers, based on the resulting volatile profiles and with the use of a number of chemometric tools. The results were also compared to unmodulated analyses (which were performed to simulate 1D‐GC‐MS analyses), which revealed that the GC×GC‐TOFMS analysis yielded up to more than 5 times the signal‐to‐noise ratios for certain analytes, in addition to enabling the detection of a higher number of compounds.^79^

### 2.2 Gas chromatography – ion mobility spectrometry

An emerging technique that is gaining increasing popularity, particularly for the analysis of food volatile flavor compounds,^14^ is GC‐IMS. When performing the SCOPUS search described in Section 2.1. with the additional search terms *ion mobility*, then 35 articles are displayed, 25 of which appeared in 2020/2021 (Figure 3; searched on 01/01/2021). Hence, more papers using GC‐IMS for this purpose have been published in 2020 alone than in all the previous years combined. A comprehensive review of the use of IMS in food analysis was published in 2019.^82^ Later a review on the use of specifically GC‐IMS and also for food flavor analysis was published in January 2020. It describes the working principles of the technique and provides a comprehensive review of all the publications where GC‐IMS were used for food flavor analysis, up until the end of 2019.^14^ The advantages of GC‐IMS include the fact that these instruments are simple, robust, low‐maintenance, and that they do not need to be operated under vacuum, which makes portability possible.^14^, ^82^, ^83^ In addition, it can be used to distinguish between isobaric compounds and certain isomers that cannot be distinguished from each other by mass spectrometry in the event that they are co‐eluted from the GC column.^82^

When a mixture of volatile compounds are analyzed using GC‐IMS, the compounds are first separated by the GC (retention time) and then in the ion mobility spectrometer (drift time), thereby adding an additional separation dimension. The retention indices (RIs) of compounds can for instance be determined relative to n‐ketones, since IMS has no response to alkanes. Subsequently compounds can be identified by comparing their experimentally determined RI and DT values to those of standards that are compiled in a GC‐IMS library.^75^, ^84^, ^85^, ^86^ However, if all of the required reference standards are not available in the laboratory and GC‐IMS is not used in conjunction with MS, it is often the case that most of the compounds in the sample cannot be positively identified.^82^ In addition, for various reasons (see later section this sections) GC‐IMS is also normally not used for the quantitation of individual compounds.^83^ Consequently, standalone GC‐IMS data is most frequently used in combination with chemometric techniques for sample classification, since using sample profiles or fingerprints for classification does not necessarily require that the identities or actual concentrations of all constituents should be known.^82^, ^87^

Therefore, a continued trend that is observed is that in many studies GC‐IMS data is used in combination with data generated using one or more additional techniques, including E‐nose,^75^, ^85^, ^88^, ^89^ E‐tongue,^13^, ^75^, ^90^ GC‐MS,^30^, ^86^, ^91^, ^92^ LC‐MS,^13^ capillary electrophoresis with ultraviolet detection (CE‐UV),^93^ fluorescence spectroscopy^84^ and proton nuclear magnetic resonance (^1^H‐NMR) spectroscopy^92^ data. In one recent report, this was called *collaborative analysis* and where both an E‐nose and an E‐tongue was used alongside HS‐GC‐IMS to evaluate the differences in the flavor of cultured pufferfish of different species. A PEN‐3 E‐nose (see the description in Section 2.1, .3.) and a TS‐5000Z E‐tongue (Insent Inc, Atsugi‐Shi, Japan) were used in this study. The sensors of this E‐tongue detects the same flavor attributes as those described in Section 2.1, .3. The GC‐IMS analyses were performed by directly injecting the headspace gasses of the samples into the GC inlet using a gastight syringe. The volatile compounds (37 in total) were identified by comparing their RI and DT values to those in a GC‐IMS library. Using PCA it could be determined that the HS‐GC‐IMS profiles of the volatile compounds can be used to clearly distinguish between four pufferfish species. PCA of the data from the E‐nose and E‐tongue analysis showed that these results are consistent with the HS‐GC‐IMS data and in particular the pufferfish species, *Takifugu rubripes*, could clearly be distinguished from the others using both of these electronic sensing techniques.^75^

**FIGURE 5:**
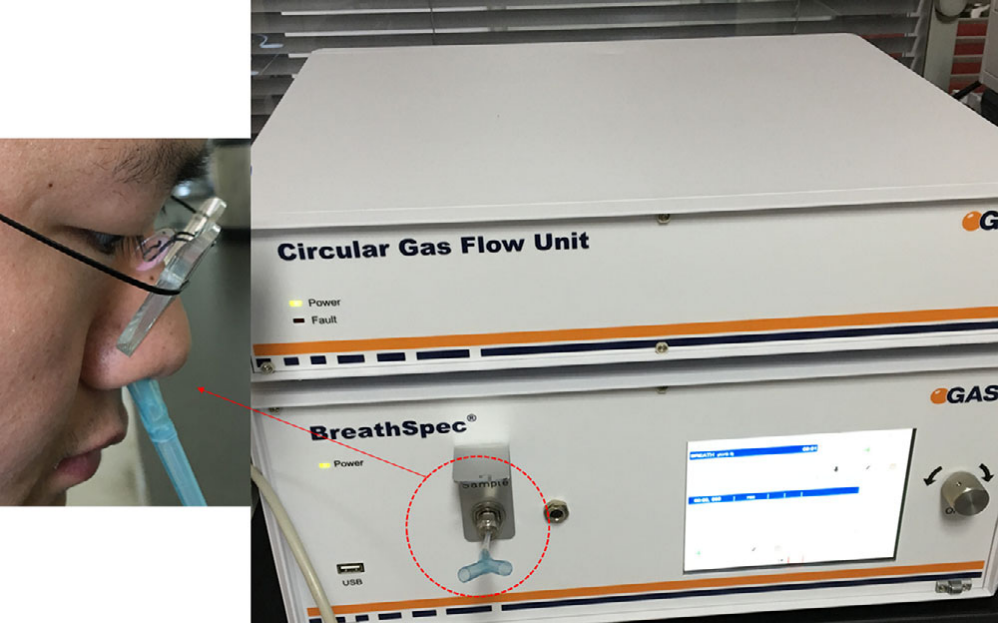
A panelist exhales through the custom made valve connected to the Breathspec GC‐IMS inlet to sample volatiles from the retronasal cavities (Image reprinted from ref. 86, Copyright (2020), with permission from Elsevier)^86^

One study of note is where GC‐IMS was not simply just used to classify samples according to their GC‐IMS fingerprint data, but instead full characterization (identification and quantitation) of the key odorants contributing to retronasal olfaction during bread consumption was performed. The GC‐IMS results were combined with results from dynamic quantitative descriptive analysis (D‐QDA) that was used to investigate the aroma that is released and the sensory perception during bread consumption. The D‐QDA was performed by trained panellists who must score (on a scale of 1–9) the intensity of six aroma attributes, namely sweet, fermentation‐like, roasty, flour‐like, creamy and sour, during defined stages of the chewing process. The volatile compounds of the bread itself and of compounds released from the retronasal cavities of the panelists were analyzed using Flavorspec HS‐GC‐IMS and Breathspec GC‐IMS instruments (G.A.S., Dortmund, Germany), respectively. In the case of the latter analyses, the breath of the panellists were analysed at each of the defined stages of the chewing process, where they had to masticate and then exhale through the nose into the specially designed valve connected to the Breathspec GC‐IMS inlet (Figure 5).^86^ The compounds were identified by comparison of their RIs and DTs to a comprehensive GC‐IMS bread database that was compiled during previous work by the same research group with the help of GC‐MS.^86^, ^94^, ^95^, ^96^ In the recent study, 31 compounds were detected by HS‐GC‐IMS, while only 24 compounds could be detected by HS‐GC‐MS. From the direct comparison of the HS‐GC‐IMS and HS‐GC‐MS results, it was revealed that a higher number of compounds were detected as well as higher signal responses could be achieved by HS‐GC‐IMS. Thus, the authors concluded that HS‐GC‐IMS is a more sensitive technique than HS‐GC‐MS for this application.^86^ Quantitative analysis was performed using the external standard approach. External standard curves of peak height vs. concentration were constructed by analyzing a series of concentrations of the relevant standards.^94^ Depending on the analyte and concentration range, either a linear curve or a curve fitted to the Boltzmann equation was obtained. It could be concluded that the perceived aroma dynamically changes during the different stages of chewing, with the sweet, creamy and roasty attributes being the most active ones. It was also determined that 3‐(methylthio)propanal, 2,3‐butanedione and acetoin are the key odour compounds contributing to the aroma.^86^

**FIGURE 6:**
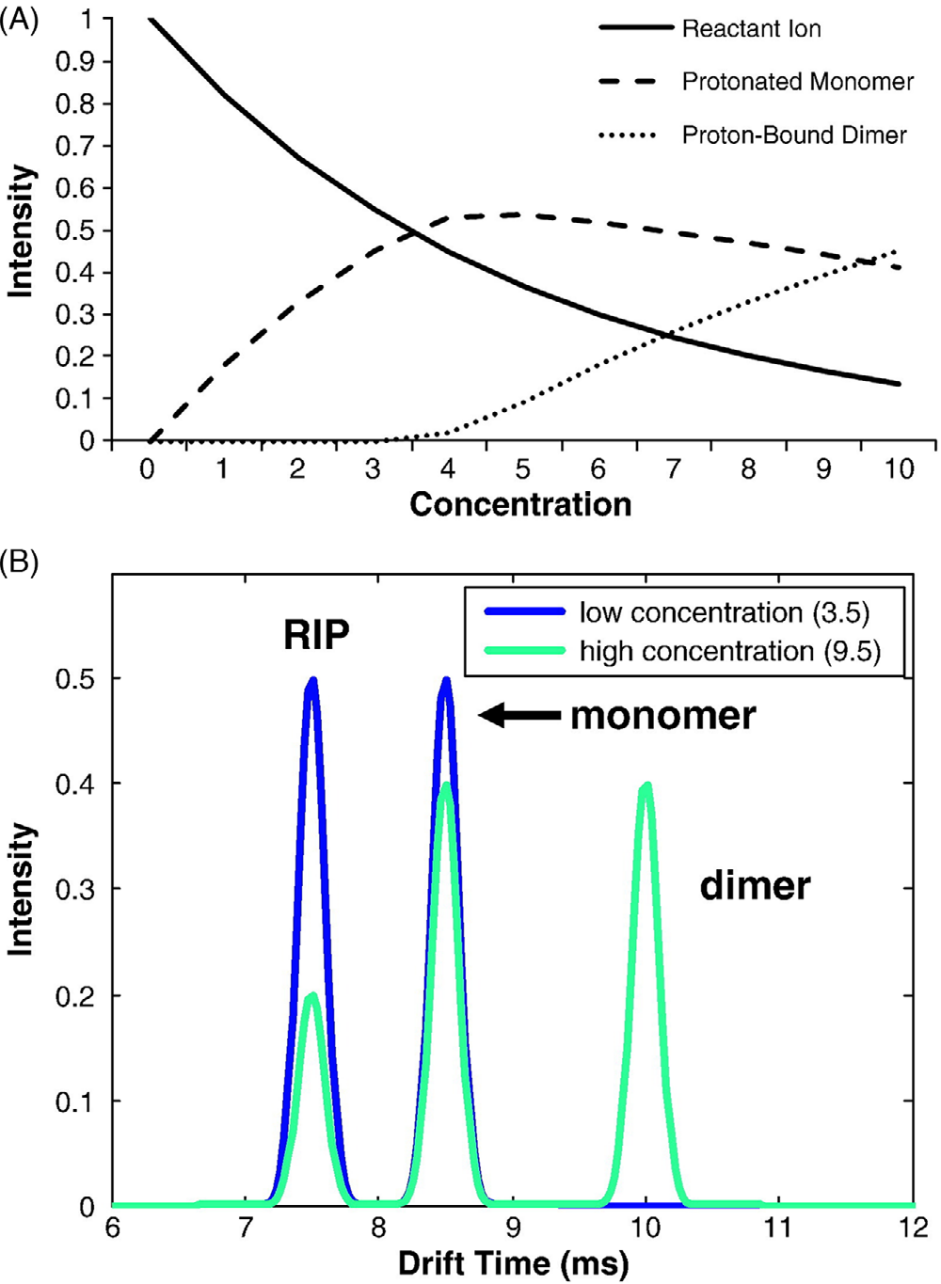
Illustration of the effect of analyte concentration on the signal intensity in IMS. (A) Signal intensity trends as concentration increases. (B) Ion mobility spectra at low and high analyte concentrations (Image reprinted from ref. 97, Copyright (2012), with permission from Elsevier)^97^

Arguably the most significant contribution in the field of GC‐IMS in 2020 is the publication by Brendel et al.,^83^ where the use of a number of different multivariate regression (MR) approaches were compared for the improved quantitation of allergenic fragrance compounds found in cosmetic products.^83^ At low analyte concentrations, the ions that are observed in ion mobility spectra are the reactant ion, i.e. proton‐water clusters (H^+^[H_2_O]_n_) and the protonated monomer of the analyte, MH^+^[H_2_O]_n‐x_. At higher analyte concentrations a proton‐bound dimer, M_2_H^+^[H_2_O]_m‐x_, is also observed (Figure 6B). The reactions in which these three ions are formed are in equilibrium. Consequently, an increase in analyte concentration initially results in an increase in the intensity of the protonated monomer peak (Figure 6A). However, once the concentration is high enough for the proton‐bound dimer to be formed, the monomer peak intensity starts to decrease gradually, instead of increasing. Once the proton‐bound dimer is formed, a similar trend is observed for its signal intensity. In addition, as the analyte concentration is increasing there is a gradual non‐linear decrease in the reactant ion peak intensity. These phenomena present a challenge when attempting to quantify analytes using IMS. When univariate regression is used to correlate the signal intensity with the analyte concentration, then the measurement of the monomer signal intensity must be performed when working at low concentrations, while the dimer signal intensity must be used when working at high concentrations. In addition, when quantifying compounds present in a complex mixture, the possibility exists that all analytes are not resolved by the GC, causing further challenges, like competitive ionization between analyte molecules and the formation of heterodimeric ions.^83^, ^97^ Therefore Brendel et al^83^ investigated the use of three MR models, namely multivariate curve resolution alternating least squares (MCR‐ALS), partial least squares regression (PLSR), and kernel‐PLSR, to quantify allergenic fragrance compounds in complex cosmetic products using non‐bilinear data generated by GC‐IMS analysis. Experiments were conducted where competitive ionization and heterodimeric ion formation scenarios were created by deliberately causing the relevant analytes to co‐elute. The results from the MR techniques were directly compared to the univariate linear regression applied to the same data. Working in a particular concentration range for all the compounds, some showed better results for the regression of their monomer signals, while for other compounds the regression of the dimer was better, when applying univariate linear regression. However, when the MR techniques were used, quantitation can reliably be performed across the entire concentration range. In particular the use of kernel‐PLSR distinctly improved quantification in both scenarios (competitive ionization and heterodimeric ion formation). It was concluded that the application of these MR techniques to the GC‐IMS data presents reliable tools that can be used for the quantitation of volatile compounds in complex matrices.

### 2.3 Miscellaneous hyphenated and combination techniques

In addition to the techniques discussed so far, a number of miscellaneous hyphenated GC techniques and techniques used in combination with GC analysis were reported in 2020. For instance, proton transfer reaction (PTR) MS was used in combination with GC‐FID,^98^ ^1^H‐NMR was used in combination with GC‐MS and GC‐FID,^99^ as well as GC‐MS and GC‐IMS,^92^ LC‐MS was used together with GC‐IMS,^13^ as well as GC‐MS,^100^, ^101^, ^102^ CE‐UV with GC‐IMS^93^ and fluorescence spectroscopy in combination with GC‐IMS^84^ and GC‐MS.^103^ In most cases the techniques were used collaboratively in order to generate complementary data.

For instance, a combination of GC‐IMS, LC‐MS, and an electronic tongue was used for the analysis of the flavor compounds of different wild mushroom soups. This combination provided a more comprehensive characterization of the flavor compounds, since both volatile and non‐volatile flavor compounds could be determined, using GC‐IMS and LC‐MS, respectively, and the resulting data could be compared to the data produced using the electronic tongue.^13^ In another study, GC‐IMS was used to analyze the volatile flavor compounds, while GC‐MS was used to analyze the fatty acids present in the samples and^1^H‐NMR was used for the determination of water‐soluble low molecular weight compounds in order to study the effect of natural spices on certain compounds in boiled Wuding chicken during processing.^92^

In terms of miscellaneous hyphenated techniques, the use of GC coupled to an electro‐antennographic detector (EAD)^104^ and GC coupled to low energy (soft) electron ionization (EI) MS,^47^ as well as tandem hard and soft EI‐MS,^105^ were reported. GC‐EAD was used to study the electrophysiological responses of Queensland fruit fly females to fruit odors. The aim of the study was to develop attractants for female flies using odors from certain host fruits. The volatile fragrance compounds of the different fruits were sampled using DHS. Then the samples were analyzed using a GC equipped with both an FID and an EAD, the latter fitted with fruit fly antennae. A total of 41 compounds were detected with the EAD and these were then identified using GC‐MS. The behavior of the flies was subsequently studied by exposing them to a selection of the EAD‐active compounds, using a Y‐tube olfactometer. Unfortunately, no correlation could be found between the behavioral and electrophysiological responses.^104^

In a study of the key odourants of Arabica coffee, SAFE and SPME were again used as complementary techniques for the extraction of the volatile aroma compounds prior to GC‐MS analysis. However, a number of the trace odourants detected using GC‐O and AEDA could not be quantified properly using GC‐MS. Therefore, a GC‐QTOF with its electron ionization (EI) source operated at a low ionization energy (15 eV) was used in order to increase the intensity of the molecular ion peaks of the trace odourants. Indeed, this approach facilitated the reliable quantitation of these analytes, by improving their detection limits.^47^

In another example, GC×GC‐TOFMS utilizing a tandem hard (70 eV) and soft (12 eV) EI technique was used to study the primary metabolites of hazelnuts. During the analysis the energy is switched from high to low at regular intervals, thereby continuously generating two different spectra for each analyte during a single analysis. The mass spectra generated using high energy produce the regular EI mass spectra that can be compared to MS databases etc. for compound identification, while the low energy conditions are used to generate spectra that provide complementary information as well as improved signal intensity of some relevant ions of certain compounds. In short, the methoxime/trimethylsilyl derivatives of the primary metabolites that were extracted from the hazelnuts were analysed using Tandem EI, which provided two significantly different mass spectra for each metabolite (hence improving confidence in their identities) as well as improved sensitivity for some metabolites at low energy ionisation. The sample fingerprints generated in this way could be used to distinguish between different hazelnut cultivars as well as their geographical origins, by means of statistical analysis. The volatile aroma compounds of the hazelnuts were also characterised using HS‐SPME‐GC‐MS. Similarly, key aroma compounds and potent odourants identified using HS‐SPME‐GC‐MS could be correlated with known non‐volatile aroma precursors that were analysed by GC×GC‐TOF‐Tandem EI‐MS.^105^

### 2.4 Novel stationary phases used for flavor and fragrance analysis

In a recent review the use of ionic liquids as GC stationary phases for food and natural product analysis was discussed in detail, including exploiting their peculiar selectivity, their orthogonality for multidimensional GC and the use of water‐compatible ionic liquids for the analysis of aqueous samples.^106^ Cagliero et al.^107^ investigated the viability of using the phosphonium‐based ionic liquid, trihexyl(tetradecyl)phosphonium chloride, routinely as a gas chromatography stationary phase. The performance of a number of columns with different lengths, internal diameters, and stationary phase film thickness was evaluated by analyzing the Grob test mixture, and the column stability and maximum allowable operating temperature was determined. The latter was found to be relatively low, that is, 210°C. The columns were subsequently tested using a mixture of 41 common flavor and fragrance compounds and a fatty acid methyl esters (FAMES) mixture and then they were applied to the analysis of essential oils. It could be concluded that the trihexyl(tetradecyl)‐phosphonium chloride stationary phase offers unique selectivity, with pronounced retention of polar analytes. Although the low maximum allowable operating temperature of 210°C can be a limitation, a suitable combination of certain parameters, including column characteristics and operating conditions could be used the facilitate the analysis of medium to low volatile FAMES. The suitability of this stationary phase to be used for essential oil analysis was also demonstrated.^107^

Another study by the same research group was performed on ionic liquid GC stationary phases that are water‐compatible. The use of ionic liquid stationary phases with phosphonium and imidazolium derivative cations in combination with trifluoromethanesulphonate for the direct analysis of fragrances and essential oils in aqueous media using GC–MS, were investigated. First, the GC‐MS conditions were optimized for the direct injection of aqueous solutions, by analyzing a mixture of fragrance allergens, dissolved either in cyclohexane or in EtOH:H_2_O (1:1). Columns coated with the two different stationary phases were evaluated separately. It was found that poor reproducibility was achieved for the analysis of the EtOH:H_2_O solutions, compared to the cyclohexane solutions. This problem was solved by using a higher than normal GC inlet pressure, which resulted in greatly improved repeatability. The optimized method was subsequently successfully applied to the analysis of aqueous‐based commercial perfumes and it was found that the two columns provide complementary selectivity. Ultimately, it was shown that these columns can be used routinely for the direct analysis of aqueous perfumes, thereby eliminating the need for time‐consuming sample preparation procedures.^108^

Then finally, in a very unique contribution, the supercritical fluid chromatography‐mass spectrometry (SFC‐MS) analysis of volatile compounds was made possible by using a newly developed highly cross‐linked styrene divinylbenzene (SDVB) polymer‐based column. Using this column it was possible to retain compounds like certain esters and non‐polar terpenes that could not be retained on regular silica‐based columns (C_18_, phenyl, and NH_2_). The authors demonstrated that volatile compounds with a wide range of chemical properties can successfully be analyzed by SFC using the new SDVB column.^109^

## 3 CONCLUSIONS AND FUTURE PROSPECTS

In 2020, GC‐MS remained the “gold standard” for the chromatographic analysis of volatile flavor and fragrance compounds. Furthermore, HS‐SPME and GC‐O were the most commonly used sampling technique and sensory analysis technique, respectively. The use of a few important variations of HS‐SPME, for example, Vac‐HS‐SPME, as well as other interesting static and dynamic headspace techniques were also reported in 2020. In addition, a number of emerging trends could be identified, notably the use of SAFE for extraction, GC‐IMS for volatile compound analysis, and electronic senses, that is, E‐noses and E‐tongues, for sensory analysis. In many instances, these emerging techniques were used in collaborative approaches as complementary techniques to others, including the “gold standard” techniques. In particular, the combination of SAFE with SPME provides better coverage of the investigated compounds by enabling the complementary extraction of a wider range of volatiles.

GC‐IMS proves to be a suitable alternative to GC‐MS for sample classification, when the identities and concentrations of all the compounds do not have to be known. In addition, GC‐IMS could be used almost independently for the chemical characterization of certain samples, provided that sufficiently comprehensive GC‐IMS databases (with RI and DT values of standard compounds) were available, albeit that GC‐MS would initially be needed to assist in compiling the databases. In addition, the potential of GC‐IMS as a reliable quantitative analysis tool has been facilitated through the use of chemometric techniques involving MV approaches. This may enable a further increase in the use of GC‐IMS for volatile flavor and fragrance charactezisation in future. Currently, however, most laboratories specializing in volatile flavor analysis would already be equipped with GC‐MS instruments, hence GC‐IMS will most likely not replace GC‐MS as the “gold standard” anytime soon, but will rather be used as a complementary technique.

Finally, the development of novel stationary phases have shown the potential of opening up new possibilities for the analysis of volatile flavor and fragrance compounds, including the direct analysis of aqueous samples as well as expanding the use of SFC to VOC analysis.

## CONFLICT OF INTEREST

The author declares that there is no conflict of interest.

## Footnotes

1 To simplify the discussions on search metrics, all SCOPUS searches discussed in the remaining sections of this review is focused on *flavor* compounds only, since in all cases a higher number of documents are found than for *fragrance* compounds.
